# Effects of Surface Textures Created Using Additive Manufacturing on Shear Bond Strength Between Resin and Zirconia

**DOI:** 10.3290/j.jad.b5053367

**Published:** 2024-03-11

**Authors:** Kang Dai, Jiang Wu, Hai Yu, Zhe Zhao, Bo Gao

**Affiliations:** a PhD Candidate, State Key Laboratory of Oral & Maxillofacial Reconstruction and Regeneration, National Clinical Research Center for Oral Diseases, Shaanxi Key Laboratory of Stomatology, Department of Prosthodontics, School of Stomatology, The Fourth Military Medical University, Xi’an, PR China. Performed the experiment, wrote the manuscript.; b Associate Professor, State Key Laboratory of Oral & Maxillofacial Reconstruction and Regeneration, National Clinical Research Center for Oral Diseases, Shaanxi Key Laboratory of Stomatology, Department of Prosthodontics, School of Stomatology, The Fourth Military Medical University, Xi’an, PR China. Experimental design, proofread the manuscript.; c Research Associate, State Key Laboratory of Oral & Maxillofacial Reconstruction and Regeneration, National Clinical Research Center for Oral Diseases, Shaanxi Key Laboratory of Stomatology, Department of Prosthodontics, School of Stomatology, The Fourth Military Medical University, Xi’an, PR China. Experimental design, proofread the manuscript.; d Professor, School of Material Science and Engineering, Shanghai Institute of Technology, Shanghai, PR China. Conceptualization, supervision, resources.; e Professor, State Key Laboratory of Oral & Maxillofacial Reconstruction and Regeneration, National Clinical Research Center for Oral Diseases, Shaanxi Key Laboratory of Stomatology, Department of Prosthodontics, School of Stomatology, The Fourth Military Medical University, Xi’an, PR China. Conceptualization, methodology, supervision, funding acquisition.

**Keywords:** additive manufacturing, bonding, resin cements, zirconia

## Abstract

**Purpose:**

This investigation aimed to assess the impact of additive manufacturing-generated surface textures on zirconia bond strength.

**Materials and Methods:**

Zirconia samples (n = 144) fabricated using digital light-processing (DLP) technology were categorized into 6 groups according to the type of surface conditioning (group NN: no designs, no air abrasion; group NY: no designs, with air abrasion; group GN: groove designs, no air abrasion; group GY: groove designs with air abrasion; group HN: hexagon grid, no air abrasion; group HY: hexagon grid, with air abrasion). Composite resin cylinders were cemented to the treated zirconia surfaces with dual-curing, self-adhesive resin cement (Clearfil SA Luting). The shear bond strength (SBS) was tested after water storage for 3 days or 3 days with an additional 10,000 thermocycles.

**Results:**

The zirconia samples fabricated using DLP technology have high accuracy. The SBS of the NY, GY, and HY groups did not significantly differ after 3 days, and neither did the SBS of the NN, GN, and HN groups. The NN, NY, and HY groups exhibited reduced SBS compared to their initial values following artificial aging, while the SBS of the remaining three groups were not diminished. The GY group obtained the highest SBS value after aging.

**Conclusion:**

Printing grooves with air abrasion can improve the bond strength.

Zirconia exhibits exceptional biocompatibility and superior mechanical strength, rendering it a highly promising ceramic for dental application. However, traditional ceramic bonding methods, including acid etching followed by silane application, are not effective with zirconia, owing to its chemical inertness and the lack of a glass phase. The long-term clinical success of zirconia restorations relies on a strong, durable bond, particularly when mechanical retention of the abutment is not ideal.^30^ Hence, to enhance the bond strength, various kinds of surface treatments and luting cements have been developed. These surface treatments include grinding,^17^ airborne particle abrasion,^23^ laser irradiation,^32^ and tribochemical silica coating.^25^ Among these methods, the application of phosphate monomers, such as 10-methacryloyloxydecyl dihydrogen phosphate monomer (MDP) following air abrasion, provides successful long-term clinical bonding.^12^ Yazigi et al^37^ reported a 100% survival rate of resin-bonded zirconia ceramic restorations for up to 12 years.

Laser irradiation is an alternative surface modification technique that utilizes ablation and vaporization to remove surface particles, thereby producing regular patterns rather than random surface roughness. Zirconia ceramic can be treated with this method to generate well-designed grooves, dimples, and grids. The roughness of surfaces textured with an Er:YAG laser was comparable to that of surfaces treated with air abrasion.^2^ Ruja et al^29^ created patterns of dots or lines on zirconia surfaces using ultrafast laser. Both patterns considerably increased microtensile bond strengths. Baersch et al^3^ created microgrooves using a femtosecond laser, which increased the bond strength by approximately 30% compared with the unconditioned surfaces and 21% compared to the sandblasting treatments. Akay et al^1^ processed zirconia ceramic with an ultrafast laser to generate a variety of surface patterns, including holes, grooves, and grids. When compared to the untreated samples, all of these showed noticeably higher flexural bond strength.

Although several advantages of laser surface texturing have been reported, laser irradiation has some challenges. Thermal cracks generated during the process may damage the mechanical properties of zirconia.^8^ Additionally, laser irradiation can induce phase transformation,^28^ which is detrimental to the long-term performance of zirconia restorations. Furthermore, for laser surface texturing, the laser beam direction should typically be perpendicular to the surface. However, this is not easy to maintain due to the complex geometry of the dental restorations, especially that of their inner surfaces. Therefore, reproducing uniform surface textures is challenging, which decreases the effectiveness of the intended function.^7^

Recently, additive manufacturing (AM) has been introduced as a highly precise method in the field of dentistry. Its manufacturing philosophy allows flexible preparation of extremely accurate and complex structures, which is not easy to achieve using conventional fabrication techniques such as casting and milling. Digital light processing (DLP), one of the AM technologies, has the ability to produce ceramic components with remarkable precision and advantageous mechanical properties.^9^ Therefore, inspired by the favorable outcomes observed in laser surface texturing, this investigation aimed to study the effects of DLP-generated surface textures on zirconia bond strength, and compare them with air-abrasion treatment. The hypothesis was that the bond strength of resin to zirconia would be improved by surface textures produced with DLP technology.

## Materials and Methods

### Material and Fabrication Processing

A 3 mol% yttrium-stabilized zirconia (3Y-TZP) suspension (CP-TZP-010, Jiaxing CeramPlus Technology; Jiaxing, China) with 50 vol% solid load was used. The suspension was then loaded into a DLP printer (DLP-Desktop, Jiaxing CeramPlus Technology; Jiaxing City, China), which cured the slurries to solidify layer by layer. The light intensity, exposure time, and printing layer thickness were set as 8 mW/cm^2^, 4 s, and 30 μm, respectively.

Models with STL format files were created using SolidWorks 2021(Dassault Systems; Concord, MA, USA). After processing, the printed green bodies were put in a furnace (KF1700, Nanjing Boyuntong Instrument Technology; Nanjing, China) with a slow debinding process up to 500°C to remove organics, then sintered at 1550°C for 2 h to obtain fully densified specimens.

### Specimen Preparation

**Fig 1:**
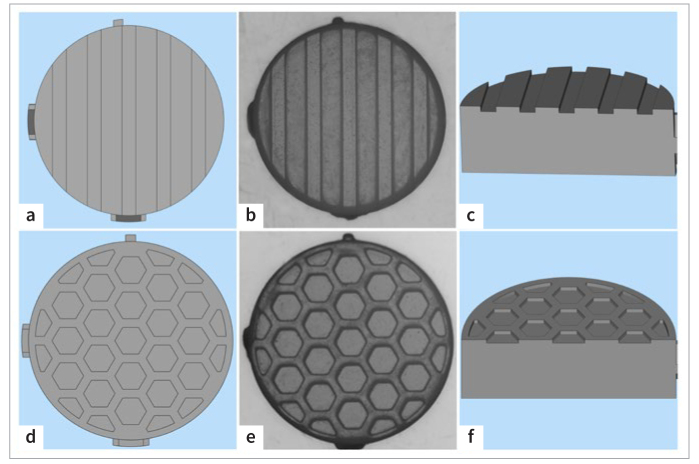
Computer-aided designs and corresponding sintered structures. (a) and (d) top view of the computer-aided designs. (b) and (e) sintered structures. (c) and (f) cross section of the computer-aided designs.

A total of 144 cylindrical (6 mm in diameter and 2 mm in thickness) Y-TZP ceramic specimens were fabricated using DLP technology. The models had two types of surface designs: groove and hexagon grid. Figure 1 shows the computer-aided designs and sintered structures. The width and depth of the groove were 0.4 mm and 0.09 mm, respectively, whereas the side length and depth of the regular hexagon grids were 0.4 mm and 0.09 mm, respectively. Specimens without surface designs served as the control group. Printing accuracy was evaluated using the KEYENCE Image Dimension Measurement System (LM-1000, Keyence; Osaka, Japan).

Based on the type of surface designs and whether or not air abrasion was performed, the specimens were categorized into 6 groups. Table 1 shows the abbreviations of the test groups and surface conditioning methods.

For airborne-particle abrasion, the zirconia disks were treated with 50-μm Al_2_O_3_ at a pressure of 0.2 MPa for 20 s. The distance between the tip of the abrasion unit (Basic Classic, Renfert; Hilzingen, Germany) to the specimen surface was 10 mm. Following ultrasonic cleaning in 99% ethanol for 10 min, all specimens were air dried.

**Table 1:** Testing groups with respect to the types of design and air abrasion

Code	Surface design	Air abrasion
NN	No	No
NY	No	Yes
GN	Groove	No
GY	Groove	Yes
HN	Hexagon grid	No
HY	Hexagon grid	Yes

Nylon molds with an inner diameter of 4 mm and a height of 2 mm were filled with a light-cured composite resin in A3 shade (Filtek Z250XT, 3M Oral Care; St Paul, MN, USA). A total of 144 cylinders were light cured (LED.F, Woodpecker; Guilin, China) for 10 s at a constant intensity of 1000 mW/cm^2^. A layer of dual-curing, self-adhesive resin cement (Clearfil SA Luting, Kuraray; Osaka, Japan, hereafter referred to as SA) containing MDP was then applied, and resin cylinders were directly bonded to the zirconia disks while being subjected to a 10 N load. Excess was extruded under loading, then removed. Subsequently, the top surface and two diametrically opposed sides of the cements were each light cured at an intensity of 1000 mW/cm^2^ for 20 s. The details of the materials used in the study are listed in Table 2.

**Table 2:** Materials employed in the current work

Material/trade name	Main composition	Manufacture
Y-TZP	ZrO_2_: 94.4 wt%, Y_2_O_3_: 5.4 wt%	Jiaxing CeramPlus, China
Filtek Z250 XT	Bis-EMA, UDMA, PEG-DMA, TEG-DMA, bis-GMA, zirconia/silica filler	3M Oral Care; St Paul, MN, USA
Clearfil SA Luting	Paste A: bis-GMA, TEG-DMA, MDP, hydrophobic aromatic dimethacrylate, dilanated barium glass filler, dilanated colloidal silica, dl-camphorquinone, benzoyl peroxide, initiators Paste B: bis-GMA, hydrophobic aromatic dimethacrylate, hydrophobic aliphatic dimethacrylate, silanated barium glass filler, silanated colloidal silica, surface-treated sodium fluoride, accelerator, pigments	Kuraray; Osaka, Japan

Bis-EMA: ethoxylated bisphenol A dimethacrylate; bis-GMA: bisphenol A glycidyl dimethacrylate; PEG-DMA: poly (ethylene glycol) dimethacrylate; TEG-DMA: triethylene glycol dimethacrylate; UDMA: urethane dimethacrylate.

### Shear Bond Strength Testing

Each bonded subgroup (n = 24) was in turn further divided into two subgroups (n = 12). One subgroup was stored in water at 37°C for 3 days without thermocycling, and the other subgroup was stored in water at 37°C for 3 days, interrupted by 10,000 thermocycles between 5°C and 55°C with a dwell time of 60 s.^5,16^ Subsequently, the zirconia portion of the specimens was embedded in self-curing acrylic resin, leaving the composite resin and adhesive interface exposed. The SBS test was performed at a crosshead speed of 0.5 mm/min with a universal testing machine (AGS-10kNG, Shimadzu; Tokyo, Japan). The SBS (in MPa) of each specimen was calculated by dividing the failure load (N) by the bonded area (mm^2^).

The effects of surface conditioning methods and aging conditions were investigated using two-way ANOVA and least significant-difference tests after the datasets met the assumptions of normality and homogeneity of variance. The data were statistically analyzed using SPSS software (IBM; Armonk, NY, USA) at a significance level of α = 0.05.

### Surface Morphology and Cross-sectional Structure

The zirconia surface and the junction between zirconia and cement were analyzed using SEM (S-4800, Hitachi; Tokyo, Japan).

### Failure Mode

To determine the failure mode, the debonded specimens were examined using an optical microscope (Nikon C-DSS230, Nikon; Tokyo, Japan). The failure types were classified as adhesive (separation in the adhesive interface), cohesive (fracture within the resin), and mixed (combination of adhesive and cohesive failure modes).

### Structures of Cement in Different Surface Designs

The structure of the cement in different surface designs was simulated using SolidWorks 2021 (Dassault Systems; Concord, MA, USA).

## Results

### Measurement of Printing Accuracy

**Fig 2:**
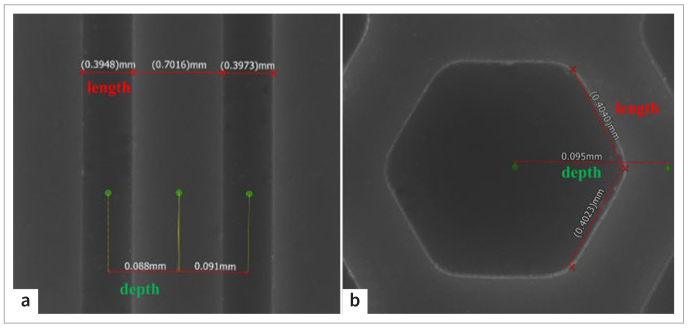
Measurement of printing accuracy. (a) Measurement of grooves. The designed width and depth were 0.4 mm and 0.09 mm, respectively. (b) Measurement of the hexagon grid. The designed side length and depth were 0.4 mm and 0.09 mm, respectively.

Figure 2 displays the measured findings of the sintered samples. Length is represented by values between two red crosses, while depth is represented by values between two green points. The designed width and depth of the groove were 0.4 mm and 0.09 mm, respectively, whereas the measured width and depth were 0.3948 and 0.088 mm, respectively. The designed side length and depth of the regular hexagon grid were 0.4 mm and 0.09 mm, respectively, while the measured length and depth were 0.4023 and 0.095 mm, respectively. It is evident that the measured value was very close to the designed value. Therefore, the size precision achieved with DLP renders it suitable for implementation in the field of dentistry.

### SEM Examination

**Fig 3:**
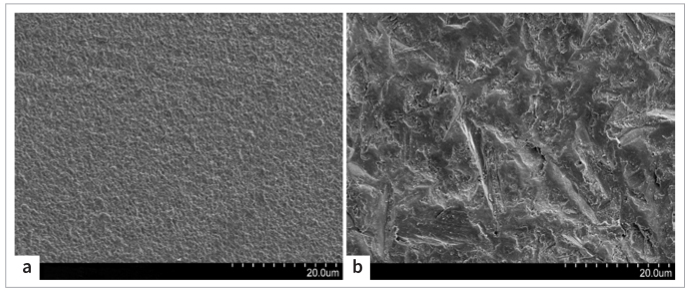
SEM of DLP-printed zirconia surface. (a) Surface morphology without air abrasion. (b) Surface morphology after air abrasion at 0.2 MPa. Original magnification 2000X.

Figure 3a shows the smooth surface of DLP-printed zirconia before air abrasion. Figure 3b exhibits the rough surface obtained after air abrasion at 0.2 MPa. The surface had irregular ridges and grooves.

**Fig 4:**
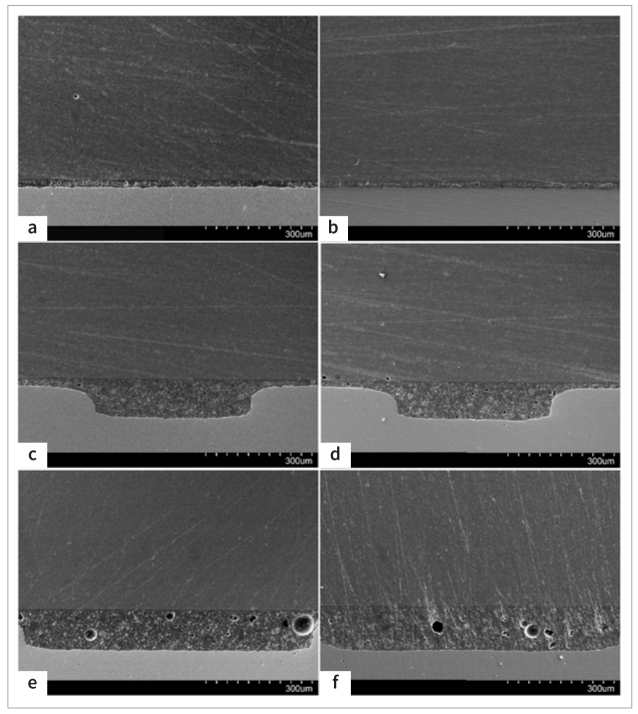
Cross section through interface. (a) NY group before aging. The upper layer is the Filtek Z250 XT resin, the middle layer is the SA luting cement, and the bottom layer is the zirconia ceramics. (b) NY group after aging. (c) GY group before aging. (d) GY group after aging. (e) HY group before aging. (f) HY group after aging. Original magnification 150X.

Figure 4 shows the cross-sectional structures of zirconia and cement before and after aging in different groups. Figure 4a reveals that the SA luting cement was well attached to Filtek Z250 XT resin and zirconia ceramics and they formed a sandwich-like structure. The thickness of the SA layer in Fig 4a is approximately 20 μm. Figures 4c to 4f shows that the luting cements infiltrated the grooves and grids. Structural defects were observed in HY group. Pores larger than 30 μm within the luting cements are presented in Figs 4e and 4f.

### SBS Results

The findings from the two-way ANOVA of the SBS testing revealed that the surface conditioning methods had a significant influence on the mean SBS (p < 0.001), whereas the aging factor did not have a significant influence (p = 0.513). Significant interactions were found between surface conditioning methods and aging conditions (p < 0.001). The SBS in each group and the corresponding statistical analysis are listed in Table 3. The SBS of the NY, GY, and HY groups did not differ statistically after three days of water storage. Meanwhile, there was no difference among the NN, GN, and HN groups. The SBS of the NY group was considerably higher than that of the NN, GN, and HN groups. After artificial aging, the SBS of the NN, NY, and HY groups were lower than their initial values, and the SBS of the GY and HN groups increased, whereas no significant difference was identified in the GN group. After aging, the GY group exhibited the highest SBS value.

**Table 3:** Mean (±SD) shear bond strengths of all groups in MPa

Group	3 days WS/0 TC Mean ± SD	3 days WS/10000 TC Mean ± SD	p-value
NN	10.3 ± 2.3^b^_A_	6.6 ± 2.1^c^_B_	0.003
NY	19.2 ± 2.9^a^_A_	16.7 ± 3.4^d^_B_	0.039
GN	9.5 ± 2.6^b^_A_	10.6 ± 2.6^e^_B_	0.353
GY	19.5 ± 3.5^a^_A_	22.9 ± 3.7^f^_B_	0.009
HN	10.2 ± 1.8^b^_A_	13.4 ± 2.8^g^_B_	0.001
HY	18.8 ± 3.3^a^_A_	16.2 ± 2.9^d^_B_	0.034

WS: water storage; TC: thermocycling; SD: standard deviation. The same superscript lowercase letters in each column indicate no statistically significant difference (p ≥ 0.05). The same subscript uppercase letters within the same row indicate no statistically significant difference (p ≥ 0.05).

### Fracture Topography Analysis

**Fig 5:**
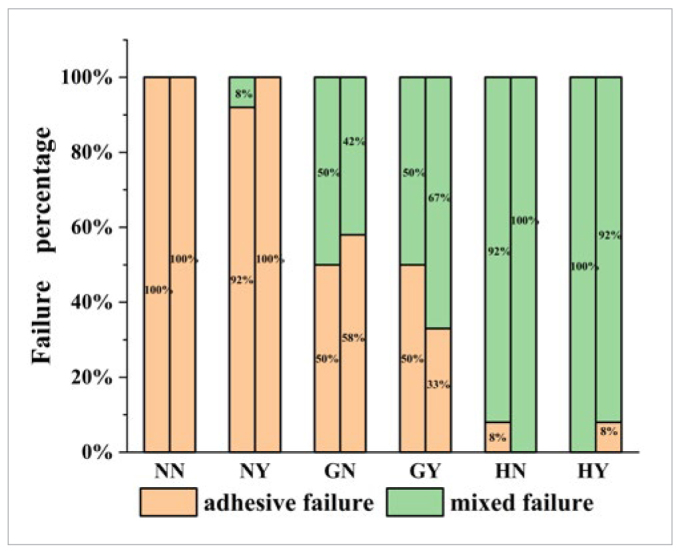
Failure mode percentage of test groups before and after aging. The first and second column of each group represents the failure mode percentage before and after aging, respectively.

**Fig 6:**
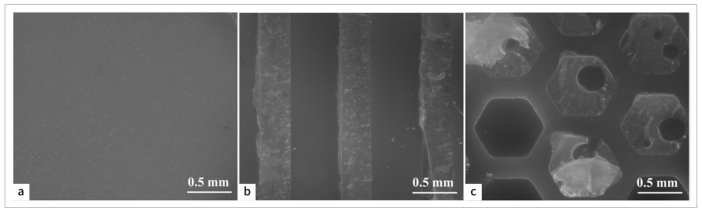
Fractured zirconia surfaces. (a) adhesive failure. (b) and (c) mixed failure. Original magnification 56X.

Figure 5 shows the failure pattern distribution for all groups after SBS testing. Almost all specimens in the NN and NY groups exhibited adhesive failure. In contrast, the percentage of mixed failure increased in the other four groups. After aging, the proportion of mixed failure in the GY and HN groups increased, while it decreased in the GN and HY groups. Figure 6 displays representative images of the fracture surfaces. It can be seen that residual resin was retained in the grooves and hexagon grids after performing the SBS test. Notably, the residual resin in the grooves was denser and more integrated than those in the hexagon grids.

### Structures of the Cements in Different Surface Designs

**Fig 7:**
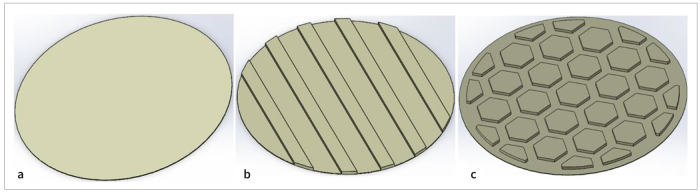
Structures of cements in different surface designs: (a) without grids, (b) with groove design, and (c) with hexagon grid design.

Figure 7 shows the simulated structures of the cement in different surface designs. It was observed that more cement was needed to fill the hexagon structure compared with the control and groove design groups.

## Discussion

At baseline storage (72 h), the initial SBS among the NY, GY, and HY groups showed no statistically significant differences. Meanwhile, there was no difference between the NN, GN, and HN groups. SEM analysis revealed that luting cement had infiltrated into the grooves and hexagon grids, generating mechanical interlocking structures (Figs 4c and 4e). However, these structures were ineffective at first, which was related to the low mechanical strength of the luting cement at the initial stage. Under the influence of light curing, the resin cement in the bonded specimen cannot fully polymerize. Lower resin cement performance is linked to a lower degree of conversion. Rizzante et al^27^ reported that immediately following light curing, resin cement only converted to a degree of about 60%, which grew progressively to 71% over the next 7 days. The findings of the SBS could be positively impacted by improved mechanical characteristics of resin cement through a greater degree of conversion.^14^ Second, the surface texture designs played a major role in the experimental results. Ruja et al^29^ found that surface lines on zirconia ceramic improved the initial microtensile bond strength; the space between the lines was only 2.5 μm, whereas the space between the grooves was approximately 700 μm (Fig 2a). Although more grooves with greater depth may improve the initial bond strength, this design can impair the strength of zirconia ceramic and lead to poor adaptation of crowns. Therefore, the depth of grooves was restricted to 90 μm, and the number of grooves was limited. Third, the bond strength of resin cements to zirconia varied significantly with different bond-strength testing methodologies (e.g., shear, tensile, and push-out).^19^ Other factors, including the variety of resins, the type of zirconia core material, and processing techniques, also affected the results.

After thermocycling, the GY group obtained the highest SBS because of post-polymerization, which enhanced the physical properties of the cement.^21,22^ The interlocking mechanism coupled with improved mechanical properties of resin cement elevated the SBS through micromechanical interaction and chemical bonding of the treated zirconia surface.^18^ In addition, MDP can chemically combine with zirconia,^34^ which is a gradual process. The production of Zr-O-P bonds increased, which contributed to the enhancement of SBS. This could explain why the SBS of the GY group increased after aging. Iwaguro et al^11^ demonstrated that the SBS of the laser surface-textured zirconia increased after 20,000 thermocycles. Yang et al^36^ observed an increase in the SBS of four different kinds of resin cement within the first month. These findings were in agreement with our study. In addition, after aging, the higher percentage of mixed failure and the fracture topography in the GY group were indicative of the strengthening effect. However, the HY group had significantly lower SBS than did the GY group after aging. The distribution of defects at the bonding interface is primarily responsible for this outcome, as large volumes of resin have more defects than small ones.^19,20^ The requirement of more cement to fill the hexagon structure often increases the probability of defect generation in the cement (Figs 4e and 4f), decreasing the measured strength after aging. Based on the above, the hypothesis that the bond strength of resin to zirconia would be improved by surface textures using DLP technology was partially accepted.

A common method to simulate the effects of aging on resin cement adherence is thermocycling. The materials experience repeated thermal expansion and contraction during this process. Zirconia and composite resin do not have the same thermal expansion coefficient, causing thermal stress that might lead to fatigue at the interface and reduce the bond strength. The number of cycles drastically varied from 500 to 37,500 cycles in different studies. It was suggested that a minimum of 5000 cycles should be applied.^20^ This investigation revealed that the SBS of the NN and NY groups decreased significantly following artificial aging, which agrees with the findings of the earlier report.^13^ This showed that the SBS might be sufficiently weakened by thermocycling for 10,000 cycles to counteract its natural increase in the controls. The SBS of the GN group, however, did not decline with aging, suggesting that the interlocking mechanism offered a potent defense against the aging impact.

Air abrasion has an important impact in terms of improving the bond strength. In the current study, the SBS of groups without air abrasion was lower than that of groups with air abrasion. SEM analysis showed that surface roughness and bonding areas were greatly improved with air abrasion. Air abrasion also increases the surface energy and reduces organic impurities, thereby increasing the wettability of the surface.^31^ Yang et al^35^ reported a remarkable reduction in bond strength without air abrasion following long-term aging, which is similar to our findings.

For a long-term durable bond, micromechanical retention as well as chemical bonding are necessary.^10^ Although many methods have been developed to promote micromechanical retention, air abrasion remains a common practice. However, the effect of air abrasion is controversial. Studies indicate that air abrasion may significantly contribute to the formation of surface defects and voids, which may compromise the strength of the ceramic and impact the reliability and clinical efficacy of restorations.^15^ Additionally, it could induce a t → m phase transformation, thereby reducing the mechanical properties of zirconia ceramic.^24^ Thus, the GN and HN groups were designed to test whether air abrasion could be replaced by printing grooves or hexagon grids. The results of the experiment showed that both before and after aging, the GN and HN groups had lower SBS than the NY group. This was caused by the cleaning and roughening effects of air abrasion, which could not be achieved by printing grooves or hexagon grids. Therefore, air abrasion with a moderate pressure of ≤0.25 MPa is suggested.^26,30^

In this study, surface-textured zirconia ceramics were successfully fabricated with a one-step process using DLP technology. The bond strength was improved by printing grooves on the zirconia ceramic surface. Compared to other traditional methods to increase the bond strength, the non-destructive nature of the treatment and the formation of a desirable net shape are two distinctive advantages of the proposed method. Additive manufacturing provides an enhanced design freedom. Different surface textures can be prepared and managed, whereas complicated geometries might be challenging and not cost-effective to produce by casting or milling methods.

As shown in this study, only two surface texture designs were evaluated. Further investigations should be conducted to determine the optimal design that can generate notable effects on improving bond strength. In addition, further research should be conducted regarding the manner in which zirconia surface textures affect its mechanical behavior, particularly its aging resistance and fatigue tolerance. Meanwhile, different luting cements may have an impact on their bonding capacity to surface-textured zirconia. These aspects should be considered in future studies.

Due to the increasing need for customized dental supplies, the dental industry is one of the most promising for AM developments. Additive manufacturing reduces manufacturing expenses and significantly increases productivity by facilitating the transition from mass production to “mass customization”. The current study yielded encouraging outcomes, demonstrating that AM technologies could address obstacles associated with dental applications, in addition to their inherent flexibility in fabricating complex structures.

A limitation of the study was the absence of an evaluation of hydrolytic durability using the current method. Prolonged water storage leads to the hydrolytic degradation of the polymer matrix and the hydrolysis of the chemical bond between zirconia and MDP.^4,6^ Wegner et al^33^ evaluated the bond durability over a water storage period of 2 years, confirming the hydrolytic durability of the two bonding systems. The short-term water storage period in the present study did not enable water saturation of the luting resin, as water diffusion into the luting interphase is a gradual process that takes months to complete. Additionally, laboratory test results require confirmation through clinical studies, which constitute the final scientific evidence regarding the efficacy of the methods employed.

## Conclusion

DLP technology was successfully used to produce highly precise surface-textured zirconia ceramics. The SBS analysis verified a novel technique for enhancing resin-to-zirconia bond strength. This study found that 1. compared to air abrasion, printing grooves or hexagon grids without air abrasion did not result in an increase in bond strength; 2. the bond strength was enhanced by a 0.09-mm-deep groove design with air abrasion.

## Clinical relevance:

Aside from their ability to precisely and flexibly realize complex structures, AM technologies have the potential to overcome challenges related to dental applications.
